# VvWRKY5 positively regulates wounding-induced anthocyanin accumulation in grape by interplaying with VvMYBA1 and promoting jasmonic acid biosynthesis

**DOI:** 10.1093/hr/uhae083

**Published:** 2024-03-25

**Authors:** Zhen Zhang, Cui Chen, Changyue Jiang, Hong Lin, Yuhui Zhao, Yinshan Guo

**Affiliations:** College of Horticulture, Shenyang Agricultural University, Shenyang 110866, China; College of Horticulture, Shenyang Agricultural University, Shenyang 110866, China; College of Horticulture, Shenyang Agricultural University, Shenyang 110866, China; College of Horticulture, Shenyang Agricultural University, Shenyang 110866, China; College of Horticulture, Shenyang Agricultural University, Shenyang 110866, China; College of Horticulture, Shenyang Agricultural University, Shenyang 110866, China; National & Local Joint Engineering Research Center of Northern Horticultural Facilities Design and Application Technology (Liaoning), Shenyang 110866, China

## Abstract

Wounding stress induces the biosynthesis of various secondary metabolites in plants, including anthocyanin. However, the underlying molecular mechanism remains elusive. Here, we reported that a transcription factor, VvWRKY5, promotes wounding-induced anthocyanin accumulation in grape (*Vitis vinifera*). Biochemical and molecular analyses demonstrated that wounding stress significantly increased anthocyanin content, and VvMYBA1 plays an essential role in this process. VvWRKY5 could interact with VvMYBA1 and amplify the activation effect of VvMYBA1 on its target gene *VvUFGT*. The transcript level of *VvWRKY5* was notably induced by wounding treatment. Moreover, our data demonstrated that VvWRKY5 could promote the synthesis of jasmonic acid (JA), a phytohormone that acts as a positive modulator in anthocyanin accumulation, by directly binding to the W-box element in the promoter of the JA biosynthesis-related gene *VvLOX* and enhancing its activities, and this activation was greatly enhanced by the VvWRKY5-VvMYBA1 protein complex. Collectively, our findings show that VvWRKY5 plays crucial roles in wounding-induced anthocyanin synthesis in grape and elucidates the transcriptional regulatory mechanism of wounding-induced anthocyanin accumulation.

## Introduction

Anthocyanins, a group of secondary metabolites distributed widely in the plant kingdom, play essential roles in plant growth and development, including coloration [1], alluring pollinators and seed dispersers [2], and acting as antioxidants or signaling molecules to protect plants from abiotic and biotic stresses [3–5]. Because of these multiple biological functions, anthocyanins are often referred to as ‘nature’s Swiss army knife’ [6].

The anthocyanin biosynthesis pathway is an extension of the flavonoid biosynthesis system, which is plant-conserved [7]. Anthocyanins are synthesized in the cytosol with catalysis by a series of enzymes, involving chalcone synthase (CHS), chalcone isomerase (CHI), flavanone-3-β-hydroxylase (F3H), dihydroflavonol-4-reductase (DFR), anthocyanidin synthase (ANS), and UDP Glc-flavonoid-3-O glucosyltransferase (UFGT) [8, 9]. UFGT, as the last step in this biosynthesis process, is critical for anthocyanin water solubility and stability [9, 10]. In addition, in many higher plants, the genes encoding the above-mentioned catalytic enzymes are transcriptionally modulated by a ternary complex known as MYB-bHLH-WD40 (MBW). This complex is composed of MYB transcription factors (TFs), basic helix–loop–helix (bHLH) TFs, and WD40 subunits [11, 12]. In apple, MdMYB1 has been reported to control anthocyanin synthesis in fruit flesh by upregulating the transcription of *MdUFGT* and *MdDFR* [13, 14]. In pear, PyMYB114 together with PyMYB10 have been shown to increase anthocyanin content in some pear cultivars by activating the transcription levels of *PyDFR* and *PyUFGT*, respectively [15, 16]. In grapevine, VvMYBA1 has been demonstrated to promote anthocyanin synthesis by directly promoting transactivation of the *VvUFGT* promoter [10, 17, 18].

Anthocyanin synthesis is also modulated by phytohormones. For example, high levels of auxin can decrease anthocyanin accumulation by inhibiting the transcription of structural and regulatory genes [19], and brassinosteroids (BRs) treatment suppresses the biosynthesis of anthocyanin and proanthocyanidin in red-fleshed apples [20]. Abscisic acid (ABA) treatment increases the anthocyanin content in grape skin, and the application of ABA considerably improves grape color qualities [21]. Furthermore, jasmonic acid (JA) modulates anthocyanin production by regulating the stability and activity of the MBW complex [22]. Exogenous methyl jasmonate (MeJA) treatment promotes anthocyanin biosynthesis in grape berries by modulating a VvmiR156-VvSPL9 module in the color conversion process [23]. Many investigations have found that JA is extensively involved in many different kinds of physiological processes in plants, such as pollen maturation, root growth, stem volume, tissue wounding, stress tolerance, anthocyanin biosynthesis, and fruit ripening [24–27]. To date, the core biosynthetic pathway of JA has been relatively well characterized [28, 29]. Upon release from the galactolipids of chloroplast membranes by phospholipase, α-linolenic acid (α-LA) is transformed into the *cis*-(+)-12-oxo-phytodienoic acid (*cis*-OPDA) intermediate by a group of enzymes, including 13-lipoxygenase (LOX), allene oxide synthase (AOS), and allene oxide cyclase (AOC). In the peroxisome, *cis*-OPDA is further reduced and β-oxidized to JA by OPDA reductase 3 (OPR3), OPC-8:0-CoA ligase 1 (OPCL1), and β-oxidation cycle enzymes. Finally, JA is modified and converted into many derivatives in the cytosol, such as JA-isoleucine (JA-Ile) [30–33].

In addition to internal factors, anthocyanin production is also mediated by diverse environmental stimuli, including temperature, drought, pathogens, nutrients, and wounding. It has been previously proven that cold and drought stresses can boost the accumulation of anthocyanins to reduce damage [34, 35]. It has also been reported that grapevine VabHLH137 contributes to defense-induced proanthocyanidin and anthocyanin synthesis to enhance tolerance to *Colletotrichum gloeosporioides* [36]. Other studies have shown that double-knockout mutant *pap1-D*/*fls1* plants accumulate large amounts of anthocyanins and exhibit improved resistance to osmotic stress [37]. Moreover, the anthocyanin content was greatly increased in rust-infected symptomatic apple tissue, and the transcription levels of *McCHS*, *McDFR*, *McANS*, *McFLS*, *McMYB10*, *McNCED*, and *McLOX* were also upregulated [38]. Furthermore, cold stress strongly induces anthocyanin production in purple head Chinese cabbage by triggering the upregulation of *BrMYB2* and *BrTT8*, which are crucial for the co-activation of the expression of anthocyanin structural genes [39]. However, there are few reports on wounding-regulated anthocyanin biosynthesis.

WRKY proteins are plant-specific TFs containing one highly conserved WRKY DNA binding domain (WRKYGQK) in their N-terminal sequence. WRKY TFs can selectively identify and bind to the W-box (TTGACC/T) elements in the promoter region of downstream genes to regulate their transcription [40]. The WRKY TF family is one of the largest families of regulatory proteins, and thus far, 70 members have been found in Arabidopsis [41], 104 members in rice [42], and 59 members in grapevine [43, 44]. WRKY TFs have been reported to play important roles in plant growth and metabolism, including seed germination [45], leaf senescence [46], stress responses [47], and anthocyanin biosynthesis [48, 49]. For instance, the WRKY proteins Arabidopsis TTG2 and Petunia PH3 can regulate flavonoid synthesis through their interactions with the MBW complex [48, 50], and VvWRKY26, a homologous gene of AtTTG2 and PhPH3, functions as a positive modulator in flavonoid accumulation in grape berries [51]. Arabidopsis AtWRKY75, apple MdWRKY41, and *Brassica napus* BnWRKY41–1 act as negative modulators in anthocyanin accumulation [49, 52, 53]. PyWRKY26 is reported to interact with PybHLH3 to co-target the *PyMYB114* promoter, thereby contributing to anthocyanin deposition in red-skinned pear [54]. In addition, heterodimer formation between VvWRKY40 and VvMYB15 synergistically promoted anthocyanin biosynthesis in grape fruits by enhancing each other’s activation on the *VvF3*′*5*′*H* and *VvUFGT* transcription [55]. However, little has been reported on how WRKY TFs respond to abiotic stress-induced anthocyanin accumulation.

In the present study, we identified that VvWRKY5, a wounding-responsive gene, can greatly promote wounding-mediated anthocyanin synthesis in a VvMYBA1-dependent manner in grape. On the one hand, VvWRKY5 interacted with VvMYBA1, a key promoter of anthocyanin synthesis, and enhanced VvMYBA1 binding to its target genes, enhancing anthocyanin accumulation. On the other hand, VvWRKY5 could promote the biosynthesis of JA, a phytohormone that acts as a positive modulator of anthocyanin accumulation, by directly activating the transcription of *VvLOX*, and this activation was enhanced by the VvWRKY5-VvMYBA1 protein complex. Altogether, our findings provide novel insights into the functions of WRKY TFs in wounding-induced anthocyanin synthesis.

## Results

### VvMYBA1 plays a positive role in wounding-induced anthocyanin production in grape

**Figure 1:**
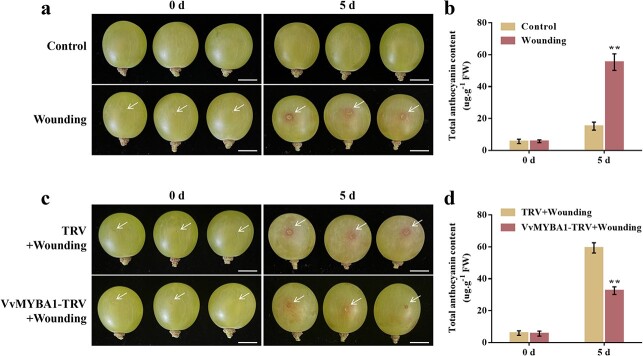
Role of VvMYBA1 in wounding-induced anthocyanin synthesis. **a** Effects of wounding treatment on anthocyanin production in grape fruits. Control, ‘Red Globe’ grape fruits without wounding; Wounding, the equatorial side of ‘Red Globe’ grape fruits was treated with a wooden stick, and the fruit stalk was wrapped with absorbent cotton to reduce water loss. Scale bar, 1 cm. **b** Total anthocyanin content in wounded grape fruits. **c** Effect of silencing *VvMYBA1* on wounding-induced anthocyanin accumulation. TRV: ‘Red Globe’ grape fruits were injected with an empty TRV before being treated with a wooden stick; VvMYBA1-TRV: ‘Red Globe’ grape fruits were injected with VvMYBA1-TRV before being treated with a wooden stick. Scale bar, 1 cm. **d** Total anthocyanin content in TRV and VvMYBA1-TRV ‘Red Globe’ grape fruits after wounding treatment. Data are means ± SDs of three separate experiments. Statistical significance at *P* < 0.01 (**) was detected using Tukey’s test.

To evaluate the impact of wounding on anthocyanin production in grape fruits, we picked ‘Red Globe’ grapes at 110 days after full bloom (DAFB), produced wounds using a wooden stick, and incubated them under light conditions. After 5 days of wounding treatment, an even redder color was observed around the wounding sites (Fig. 1a). In addition, a higher anthocyanin content was detected in wounding-treated grape fruits than in the non-wounding treatment controls (Fig. 1b). This finding indicates that wounding stress promotes anthocyanin synthesis in grape fruits and is consistent with previous studies [5, 56, 57].

VvMYBA1 is well known to be a positive modulator of anthocyanin synthesis in grape, with a crucial role in grape berry skin coloring [18, 58]. To evaluate the possible contribution of VvMYBA1 to wounding-promoted anthocyanin accumulation, a VvMYBA1-TRV (virus-mediated *VvMYBA1* inhibition) recombinant plasmid was constructed for *Agrobacterium*-mediated transient injection assays (Fig. S1, see online supplementary material), with the empty vector TRV serving as the control. After 5 days of wounding treatment, we discovered that wounding-induced anthocyanin production was dramatically reduced in the VvMYBA1-TRV grape fruits compared to the controls (Fig. 1c and d), demonstrating that VvMYBA1 plays a positive function in the anthocyanin accumulation induced by wounding treatment.

### VvWRKY5 directly interacts with VvMYBA1

**Figure 2:**
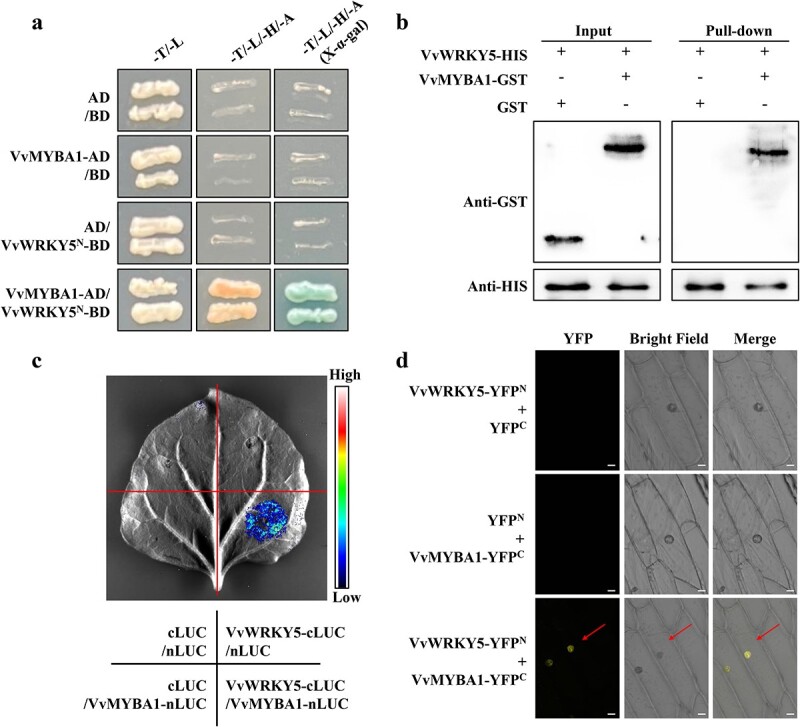
Interaction of VvWRKY5 with VvMYBA1. **a** Interaction of VvWRKY5^N^ (1–201 aa) with VvMYBA1 in Y2H assays. The negative control (NC) was the co-transformation of AD and BD. **b** Confirmation of the connection between VvWRKY5 and VvMYBA1 by pull-down assays. The protein band emphasized by the GST antibody illustrates that VvWRKY5-HIS pulled down VvMYBA1-GST. **c** Interaction of VvWRKY5 with VvMYBA1 in LCI assays. Agrobacterium strains GV3101 harboring VvWRKY5-cLUC and VvMYBA1-nLUC constructs were immersed into *N. benthamiana* leaves. After incubation in the dark for 3 days, the fluorescence signal was detected. **d** VvWRKY5 and VvMYBA1 interaction in a BiFC assay. The scale bar represents 20 μm.

WRKY-MYB module plays a crucial role in anthocyanin synthesis [53, 59]. To search for the WRKY genes in response to wounding stress in grape, we examined the expression levels of 20 WRKY genes in wounded grape fruits by RT–qPCR analysis. The results showed that three WRKY genes (*VvWRKY5*, *VvWRKY13*, and *VvWRKY24*) were significantly induced (>3-fold) by wounding stress (Fig. S2, see online supplementary material). To explore whether these three WRKY genes are involved in VvMYBA1-mediated anthocyanin accumulation under wounding treatment, we performed yeast two-hybrid (Y2H) assays. The *VvMYBA1* gene was inserted into the pGADT7 vector, whereas the *VvWRKY5^N^* (*VvWRKY5* without the self-activating fragment), *VvWRKY13*, and *VvWRKY24* were incorporated into the pGBKT7 vectors. Yeast cells were transformed with the recombinant plasmids and grown at 30°C on SD (−T/−L) medium and SD (−T/−L/−H/−A) medium. According to the Y2H results, the yeast cells co-transformed with VvMYBA1-AD and VvWRKY5^N^-BD exhibited normal growth on SD (−T/−L/−H/−A) medium (Fig. 2a), while yeast cells co-transformed with VvMYBA1-AD/VvWRKY13-BD and VvMYBA1-AD/VvWRKY24-BD could not grow normally (Fig. S3, see online supplementary material), indicating that VvWRKY5 interacts with VvMYBA1 in yeast cells. Subsequently, GST pull-down assays were performed using the VvWRKY5-HIS and VvMYBA1-GST fusion proteins. We found that the fusion protein VvMYBA1-GST could be pulled down by the VvWRKY5-HIS fusion protein, while the GST protein itself could not be pulled down (Fig. 2b), indicating that VvWRKY5 interacts with VvMYBA1 *in vitro*. Next, luciferase complementation imaging (LCI) experiments were carried out in *Nicotiana benthamiana* to prove the interaction of VvWRKY5 and VvMYBA1. As the results show in Fig. 2c, the fluorescence signal produced by VvWRKY5-cLUC+VvMYBA1-nLUC co-transformation in *N. benthamiana* leaves was stronger compared with that produced by the controls (cLUC+nLUC, cLUC+VvMYBA1-nLUC, and VvWRKY5-cLUC+nLUC). Finally, *in vivo* bimolecular fluorescence complementation (BiFC) assays illustrated that the simultaneous expression of VvWRKY5-YFP^N^ and VvMYBA1-YFP^C^ generated a stable YFP fluorescence signal in the onion nucleus, while no YFP signal was observed in the controls (Fig. 2d), further confirming the interaction between VvWRKY5 and VvMYBA1.

### *VvWRKY5* is responsive to wounding stress

**Figure 3:**
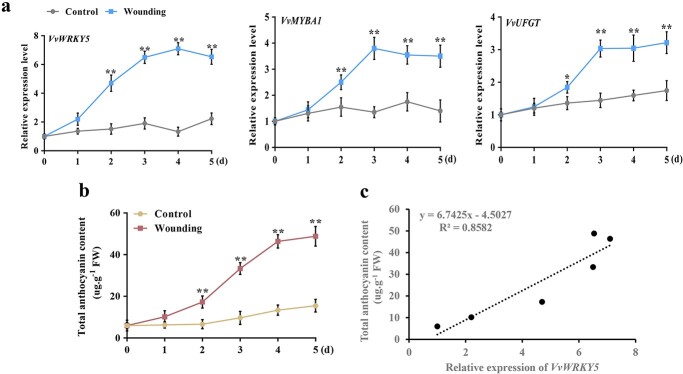
Expression profiles of *VvWRKY5* under wounding treatment. **a** RT–qPCR analysis of the expression patterns of *VvWRKY5*, *VvMYBA1*, and *VvUFGT* in grape fruits at 0, 1, 2, 3, 4, and 5 days after wounding treatment. *VvActin* was utilized as an internal control gene. Control, ‘Red Globe’ grape fruits without wounding. **b** Total anthocyanin content in grape fruits at 0, 1, 2, 3, 4, and 5 days after wounding treatment. **c** Correlation analysis of the *VvWRKY5* expression level and total anthocyanin content in grape fruits at 0, 1, 2, 3, 4, and 5 days after wounding treatment. Data are means ± SDs of 3 separate experiments. Statistical significance at *P* < 0.05 (*) and *P* < 0.01 (**) was detected using Tukey’s test.

To understand how *VvWRKY5* responds to wounding stress, we next monitored the sustained expression pattern of *VvWRKY5* in grape fruits following wounding treatment. RT–qPCR analysis showed that *VvWRKY5* expression was up-regulated upon wounding treatment, with peak transcription levels detected at 4 d, and subsequently maintained a high expression level (Fig. 3a). Interestingly, we discovered that the expression of *VvMYBA1* and its target gene *VvUFGT* was also up-regulated after wounding stress (Fig. 3a). The anthocyanin content near the wounding site also continuously increased until the end of the experiment (Fig. 3b). In addition, the correlation analysis showed that *VvWRKY5* expression was highly correlated with the total anthocyanin content (the correlation coefficient was 0.8582; Fig. 3c). Collectively, these findings imply that *VvWRKY5* may be involved in wounding-induced anthocyanin synthesis.

### VvWRKY5 contributes to wounding-induced anthocyanin synthesis in a VvMYBA1-dependent manner

**Figure 4:**
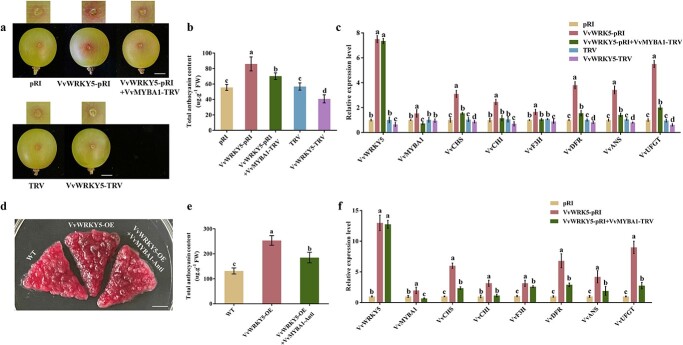
Effect of VvWRKY5 in wounding-promoted anthocyanin accumulation and genetic analysis of VvWRKY5 and VvMYBA1. **a**–**c** Appearances (**a**), total anthocyanin content (**b**), and expressions of anthocyanin synthesis-related genes (**c**) of ‘Red Globe’ grape fruits transiently expressing *VvWRKY5* after wounding treatment. VvWRKY5-pRI, transiently overexpressing *VvWRKY5*; VvWRKY5-TRV, transiently silencing *VvWRKY5*; VvWRKY5-pRI + VvMYBA1-TRV, transiently overexpressing *VvWRKY5* and silencing *VvMYBA1*. Scale bar, 1 cm. **d**–**f** Phenotypes (**d**), total anthocyanin content (**e**), and expressions of anthocyanin biosynthesis-related genes (**f**) of *VvWRKY5* transgenic grape calli. VvWRKY5-OE, *VvWRKY5*-overexpressing grape calli; VvWRKY5-OE + VvMYBA1-Anti, inhibition of *VvMYBA1* in the background of *VvWRKY5* overexpressed grape calli. Scale bar, 1 cm. Data are means ± SDs of three separate experiments. Different letters indicate statistical significance at *P* < 0.05 detected using Tukey’s test.

Given that *VvWRKY5* was responsive to wounding treatment and that its protein could interact with VvMYBA1, a positive modulator of wounding-promoted anthocyanin synthesis, we predicted that VvWRKY5 might be associated with wounding-promoted anthocyanin synthesis. To verify this hypothesis, transient injection assays were performed in ‘Red Globe’ grape fruits (110 DAFB). The VvWRKY5-pRI (*VvWRKY5* overexpression) and VvWRKY5-TRV (virus-induced *VvWRKY5* suppression) recombinant vectors were constructed and injected into grape fruits by *Agrobacterium*-mediated genetic transformation before being treated with a wooden stick. Meanwhile, empty vectors pRI and TRV were utilized as controls. The findings revealed that *VvWRKY5* overexpression significantly promoted wounding-induced anthocyanin biosynthesis; conversely, *VvWRKY5* inhibition reduced wounding-induced anthocyanin synthesis (Fig. 4a and b), which suggested that VvWRKY5 plays a positive role in wounding-induced anthocyanin synthesis. In addition, we observed that the expressions of critical genes associated with anthocyanin synthesis (*VvCHS*, *VvCHI*, *VvF3H*, *VvDFR*, *VvANS*, and *VvUFGT*) were also up-regulated in grape fruits with *VvWRKY5* overexpression and down-regulated expression in fruits with *VvWRKY5* suppression (Fig. 4c), indicating that VvWRKY5 may contribute to wounding-induced anthocyanin deposition by modulating the expression levels of genes associated with anthocyanin biosynthesis.

Interestingly, we observed that the suppression of *VvMYBA1* partially reduced the promotive effect of VvWRKY5 on wounding-induced anthocyanin deposition (Fig. 4a and b). In addition, the up-regulated transcription levels of anthocyanin biosynthesis-related genes became less significant in grapes with simultaneous overexpression of *VvWRKY5* and silencing of *VvMYBA1* (Fig. 4c).

To further reveal the connection between VvMYBA1 and VvWRKY5 in anthocyanin synthesis, we also created stable transgenic grape calli with suppressed *VvMYBA1* expression in the background of *VvWRKY5* overexpressed calli (VvWRKY5-OE + VvMYBA1-Anti). Anthocyanin accumulation experiments revealed that suppression of *VvMYBA1* significantly reduced the effect of *VvWRKY5* overexpression-promoted anthocyanin accumulation (Fig. 4d and e). In addition, the expression levels of the genes associated with anthocyanin synthesis were all significantly inhibited in VvWRKY5-OE + VvMYBA1-Anti grape calli compared to VvWRKY5-OE grape calli (Fig. 4f). Collectively, these results suggest that VvWRKY5 may promote wounding-induced anthocyanin deposition in a VvMYBA1-dependent manner.

### VvWRKY5 enhances the transcriptional activity of VvMYBA1

**Figure 5:**
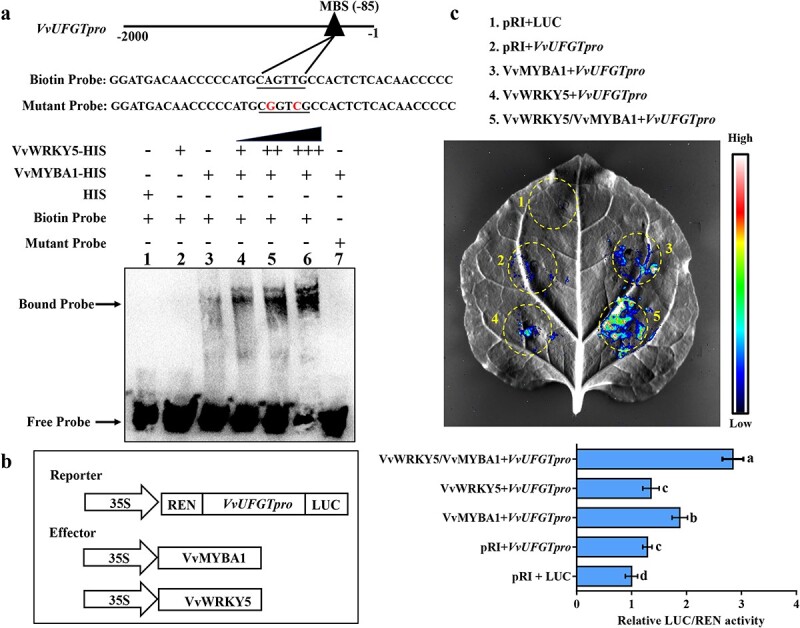
VvWRKY5 promotes *VvUFGT* transcriptional activation via VvMYBA1. **a** EMSAs demonstrated that VvWRKY5 enhances the binding capacity of VvMYBA1 to the *VvUFGT* promoter. The character ‘+’ denotes presence, while ‘−’ denotes absence. **b** Diagram of the reporter structure harboring the *VvUFGT* promoter and the effector structures harboring *VvWRKY5* and *VvMYBA1* CDSs. **c** Based on the results of dual-luciferase reporter experiments, the VvWRKY5-VvMYBA1 interaction enhanced the transcriptional activation of VvMYBA1 on the *VvUFGT* promoter. Data are means ± SDs of three separate experiments. Different letters indicate statistical significance at *P* < 0.05 detected using Tukey’s test.

As mentioned above, overexpression of *VvWRKY5* dramatically up-regulated the expressions of structural genes associated with anthocyanin synthesis and slightly increased the expression of *VvMYBA1* (Fig. 4c and f). Previous research has demonstrated that VvMYBA1 directly activates the transcription of *VvUFGT* to promote anthocyanin synthesis [18, 60]. However, the Y1H experimental results in this study indicated that VvWRKY5 did not bind directly to the promoters of *VvMYBA1* and *VvUFGT* (Fig. S4, see online supplementary material). Considering the interaction between VvWRKY5 and VvMYBA1, we hypothesized that VvWRKY5 may carry out its regulatory function by affecting VvMYBA1’s binding capacity to its target gene *VvUFGT*. To verify this speculation, we conducted electrophoretic mobility shift assays (EMSAs) using the fusion proteins VvWRKY5-HIS and VvMYBA1-HIS as well as the biotin-labeled *VvUFGT* probe containing the MBS element. The EMSAs showed that VvMYBA1 can directly bind to the *VvUFGT* probe (Fig. 5a, lane 3), while empty HIS (Fig. 5a, lane 1) and VvWRKY5 (Fig. 5a, lane 2) cannot. When the *VvUFGT* probe was mutated with two nucleotides, the shifted band disappeared (Fig. 5a, lane 7). Importantly, as more amounts of the VvWRKY5-HIS fusion protein were added, the binding affinity of the VvMYBA1-HIS fusion protein and *VvUFGT* probe was gradually increased (Fig. 5a, lanes 4–6), indicating that VvWRKY5 enhances the binding ability of VvMYBA1 to the *VvUFGT* promoter. This result was further confirmed in *N. benthamiana* leaves using dual-luciferase reporter assays with *proVvUFGT*-LUC as a reporter and 35S::*VvWRKY5* and 35S::*VvMYBA1* as effectors (Fig. 5b). Luminescence detection revealed that coexpression of 35S::*VvMYBA1* + *proVvUFGT*-LUC (Fig. 5c, coinfiltration 3) resulted in a more obvious fluorescence signal compared with the pRI + *proVvUFGT*-LUC (Fig. 5c, coinfiltration 2), while coexpression of 35S::*VvWRKY5* + *proVvUFGT*-LUC (Fig. 5c, coinfiltration 4) could not, indicating that VvMYBA1 could activate the expression of the *VvUFGT* promoter but not VvWRKY5. Furthermore, a substantially stronger luminescence signal was observed in the 35S::VvWRKY5/35S::*VvMYBA1* + *proVvUFGT*-LUC samples (Fig. 5c, coinfiltration 5) than in the 35S::*VvMYBA1* + *proVvUFGT*-LUC samples (Fig. 5c, coinfiltration 3), which further confirmed that VvWRKY5 enhances the binding capacity of VvMYBA1 to the *VvUFGT* promoter.

### JA promotes anthocyanin synthesis in grape

**Figure 6:**
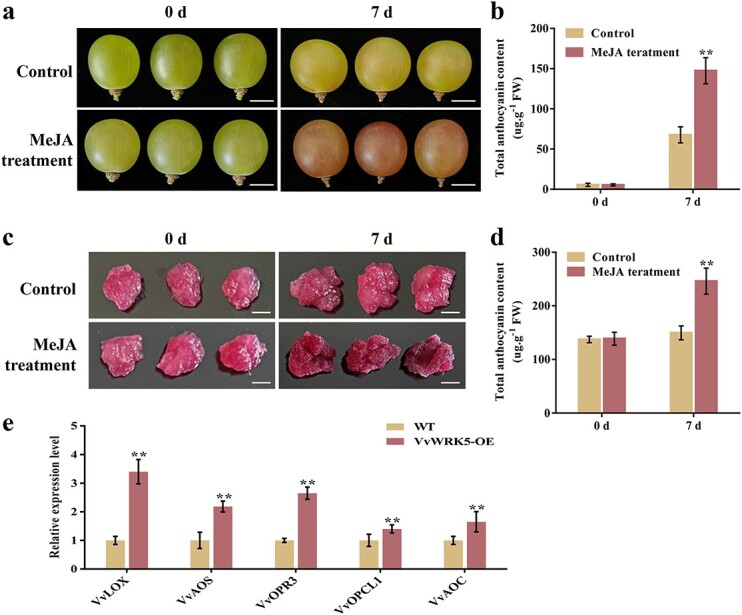
The impacts of exogenous MeJA treatment on grape anthocyanin synthesis. **a**, **b** Phenotypes (**a**) and total anthocyanin content (**b**) of ‘Red Globe’ grape fruits treated with 100 μM MeJA for 7 days. Control, grape fruits without MeJA treatment. Scale bar, 1 cm. **c**, **d** Phenotypes (**c**) and total anthocyanin content (**d**) of ‘Gamay’ grape calli cultured on a B5 media containing 100 μM MeJA. Control, grape calli without MeJA treatment. Scale bar, 1 cm. **e** Expressions of JA synthesis genes in VvWRKY5-OE grape calli. Data are means ± SDs of three separate experiments. Statistical significance at *P* < 0.01 (**) was detected using Tukey’s test.

JA has been reported to be actively involved in the modulation of plant anthocyanin accumulation [61, 62]. To investigate the effect of JA on anthocyanin biosynthesis in grape, 100 μM MeJA (a stable JA derivative) was used to treat ‘Red Globe’ grape fruits (110 DAFB). The treated grape fruits were then incubated under light conditions for 7 days. Compared with the control grapes, MeJA treatment significantly induced grape berry coloring, with increased anthocyanin content (Fig. 6a and b). Similarly, we found that MeJA treatment could promote anthocyanin synthesis in grape calli (Fig. 6c and d). In conclusion, these findings imply that JA contributes to anthocyanin accumulation in grape.

Our previous studies revealed that overexpression of *VvWRKY5* boosted endogenous JA and JA-Ile levels in grape calli [63]. Therefore, we speculate that VvWRKY5 may also promote wounding-induced anthocyanin deposition by enhancing the increased endogenous JA content. Next, we looked at the expression of genes involved in JA biosynthesis (*VvLOX*, *VvAOS*, *VvOPR3*, *VvOPCL1*, and *VvAOC*) in WT control and VvWRKY5-OE calli. We found that compared with the WT calli, the mRNA levels of JA biosynthesis genes were all significantly elevated in VvWRKY5-OE grape calli, especially *VvLOX* and *VvOPR3* (Fig. 6e).

### VvWRKY5 binds to the *VvLOX* promoter to promote its transcription

**Figure 7:**
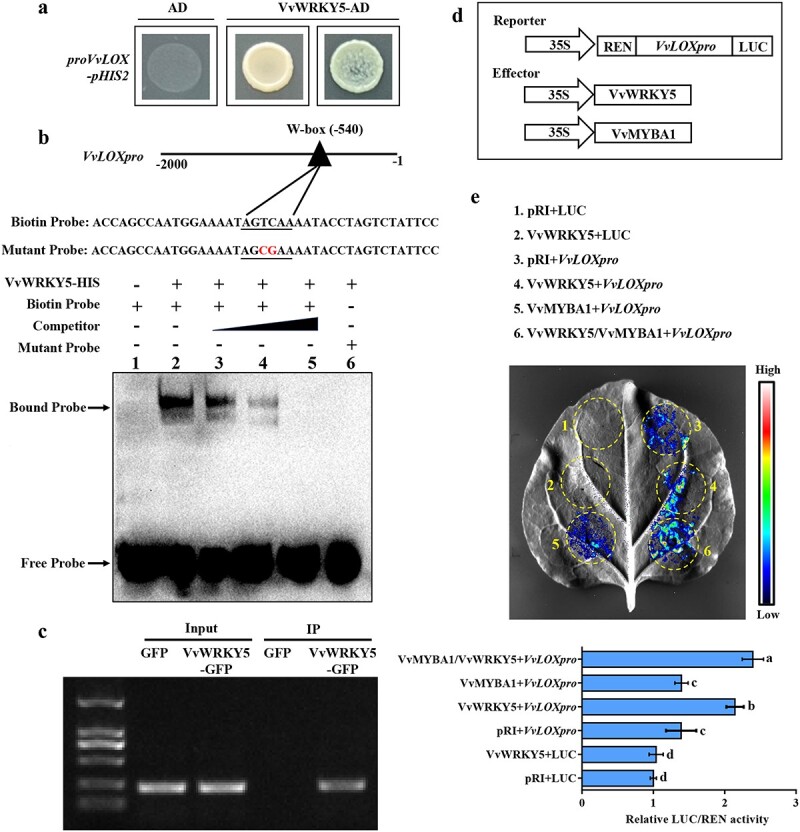
VvWRKY5 interacts with the promoter of *VvLOX* and activates its transcription. **a** Y1H experiments demonstrated the interaction between VvWRKY5 and the *VvLOX* promoter. The AD vector served as the NC. The optimal concentration of 3-AT was 160 mM. **b** EMSAs revealing that VvWRKY5 binds to the W-box site in the *VvLOX* promoter. The characters ‘+’ and ‘−’ represent presence and absence, respectively. As the competitive probe, a 200-fold excess of the non-labeled probe was used. **c** ChIP–PCR results illustrated that VvWRKY5 interacted with the *VvLOX* promoter *in vivo*. Anti-GFP antibody was utilized to precipitate cross-linked chromatin samples obtained from VvWRKY5-GFP-overexpressed grape calli. The GFP-overexpressed grape calli served as the NC. **d** Diagram of the reporter structure harboring the *VvLOX* promoter and the effector structures harboring *VvWRKY5* and *VvMYBA1* CDSs. **e** Dual-luciferase reporter experiments suggested that VvWRKY5 promotes the promoter activity of *VvLOX*, and this activation was enhanced by VvMYBA1. Data are means ± SDs of three separate experiments. Different letters indicate statistical significance at *P* < 0.05 detected using Tukey’s test.

To further investigate the molecular mechanism by which VvWRKY5 regulates JA synthesis, the interactions between VvWRKY5 and the promoters of *VvLOX* and *VvOPR3* were analysed using yeast one-hybrid (Y1H) experiments. Our findings showed that Y187 yeast cells co-transformed with VvWRKY5-AD and *proVvLOX*-pHIS2 grew normally in SD (−T/−L/−H) medium containing 160 mM 3-AT (Fig. 7a), indicating that VvWRKY5 could interact with the promoter of *VvLOX*. However, VvWRKY5 could not bind to the promoter of *VvOPR3* (Fig. S5, see online supplementary material). WRKY TFs specifically recognize W-box elements in the promoter sequences of their target genes [64]. In this work, we identified only one W-box element (−540 bp) in the *VvLOX* promoter using PlantCARE analysis. Then, we designed EMSA probes using the promoter segment containing this W-box element. The results of EMSAs confirmed that VvWRKY5 could bind to the *VvLOX* promoter (Fig. 7b, lane 2), and this binding gradually weakened or even completely suppressed as the concentration of competitor probes increases (Fig. 7b, lanes 3–5). In addition, when the *VvLOX* probe was mutated with two nucleotides, the shifted band disappeared (Fig. 7b, lane 6). Subsequently, we performed chromatin immunoprecipitation PCR (ChIP-PCR) experiments to test the binding of VvWRKY5 to the *VvLOX* promoter in grape calli. According to the ChIP-PCR results, the promoter fragment of *VvLOX* was highly enriched in VvWRKY5-OE grape calli (Fig. 7c), reflecting the *in vivo* interaction of VvWRKY5 to the *VvLOX* promoter. Collectively, these outcomes showed that VvWRKY5 specifically binds the W-box motif in the *VvLOX* promoter.

We next conducted a dual-luciferase reporter experiment to analyse how VvWRKY5 modulates *VvLOX* promoter activity. In this assay, *proVvLOX*-LUC was constructed as a reporter, while 35S::*VvWRKY5* was constructed as an effector (Fig. 7d). The LUC results revealed that a more obvious fluorescence signal was detected in *N. benthamiana* leaves coexpressing 35S::*VvWRKY5* + *proVvLOX*-LUC (Fig. 7e, coinfiltration 4) compared with the controls (Fig. 7e, coinfiltration 1–3), indicating that VvWRKY5 could directly activate the transcription of the JA biosynthesis-related gene *VvLOX*. In addition, the tobacco leaves co-expressing 35S::*VvWRKY5/*35S::*VvMYBA1* + *proVvLOX*-LUC (Fig. 7e, coinfiltration 6) produced a much stronger fluorescence signal than the individual expression of 35S::*VvWRKY5* with *proVvLOX*-LUC (Fig. 7e, coinfiltration 4), suggesting that VvMYBA1 may enhance the transcriptional activation of VvWRKY5 on the *VvLOX* promoter.

## Discussion

Wounding is one of the most important stresses on fruits and can lead to deterioration of grape quality. In particular, high-temperature and high-humidity environments are more likely to cause bacterial invasion in the wound area. Solving the problem of grape fruit wounding damage can effectively extend the storage shelf life of grapes and improve their commodity value. Anthocyanins are a type of plant secondary metabolite that plays an important function in protecting plants from external stresses [65, 66]. Numerous studies have demonstrated that anthocyanin biosynthesis is greatly increased when plants are subjected to environmental stresses [34], including cold stress [67, 68], salt stress [69, 70], drought stress [71, 72], and wounding stress [73, 74]. In apple, wounding treatment of apple fruits leads to more anthocyanin accumulation [59]. In *Rosa rugosa*, anthocyanin content increased in wounded petals [5]. In this study, we found that more anthocyanin accumulated around the wounding sites of grape fruits (Fig. 1a and b), indicating that wounding can induce anthocyanin biosynthesis in grape. However, the specific molecular mechanism underlying wounding-induced anthocyanin production is unclear.

The MBW protein complex is important for modulating anthocyanin synthesis [12, 75]. Many environmental factors that modulate anthocyanin accumulation depend on the MBW complex [13]. In previous studies, VvMYBA1 has been confirmed as a positive modulator in anthocyanin production and fruit coloration in grapes through directly promoting the transcription of the structural gene *VvUFGT* [18, 60]. In the current study, we observed that *VvMYBA1* expression was up-regulated under wounding treatment (Fig. 3a), and wounding promoted anthocyanin accumulation in grape in a VvMYBA1-dependent manner (Fig. 1), which further proved the essential role of VvMYBA1 in anthocyanin synthesis as well as in wounding-induced anthocyanin deposition.

The coordinated actions of multiple TFs are important for regulating plant growth and regulation, and MYB TFs can collaborate with other TFs to co-modulate downstream gene transcription. For example, in apple, MdMYB63 and MdERF106 interact to increase the promoter activity of *MdSOS1*, thereby improving salt tolerance [76]. MdMYB10 and MdbHLH3 interact to co-modulate anthocyanin accumulation in fruit skin, and MdMYBPA1 and MdbHLH33 interact to enhance anthocyanin biosynthesis in low-temperature environments [14, 77]. In loquat, EjMYB1/EjMYB2 have been demonstrated to work together with EjAP2–1 to promote the synthesis of lignification [78]. In our study, we verified that VvMYBA1 could interact with VvWRKY5 by four protein interaction assays (Fig. 2). In addition, the interplay between VvWRKY5 and VvMYBA1 synergistically increased the transcriptional activation of VvMYBA1 on its target gene *VvUFGT* (Fig. 5). Furthermore, expression analysis showed that *VvWRKY5* transcription was dramatically induced under wounding treatment (Fig. 3a), which ultimately results in further increases of anthocyanin content in wounded grapes. Our observation of this WRKY-MYB complex is similar to previous findings in apple that MdWRKY40 and MdMYB1 collaborate to facilitate the binding of MdMYB1 to its targets *MdDFR*/*MdUFGT* promoters [59]. It is worth noting that VvMYBA1 and VvMYBA2 are considered as a part of a single haplotype (allele), as they are densely packed in a single locus, which is known as the berry color locus [60]. Therefore, whether VvMYBA2 is involved in wounding-induced anthocyanin production and whether the VvWRKY5-VvMYBAs regulatory module is also applicable to VvMYBA2 will be our next research focus.

WRKY TFs play pivotal roles in various plant physiological events, including stress responses and anthocyanin biosynthesis [79, 80]. In our previous studies, we demonstrated that overexpression of *VvWRKY5* improved white rot resistance in grape fruits and calli [63]. In this study, we found that VvWRKY5 also works as a positive modulator in wounding-promoted anthocyanin synthesis, which expands functional research on the WRKY5 protein in grape.

Given our previous findings that the JA and JA-Ile contents were dramatically enhanced in *VvWRKY5*-overexpressing gape calli relative to the WT [63], we speculate that VvWRKY5 may also modulate wounding-induced anthocyanin biosynthesis by promoting JA biosynthesis. Notably, we found that exogenous JA treatment contributes to anthocyanin accumulation in grape (Fig. 6), which corresponds with prior studies in Arabidopsis [62], apple [81], and pear [82]. To date, there have been many reports on the molecular mechanism of JA signaling-modulated anthocyanin production. In Arabidopsis, the JA signaling receptor COI1 seems to be a critical factor in the expressions of the JA-mediated regulators (PAP1 and PAP2) and JA-induced anthocyanin synthesis. In addition, JAZ proteins are regarded as negative modulators of the JA response and anthocyanin biosynthesis. JA stimulates the degradation of JAZ proteins to release MYC2, thereby promoting anthocyanin biosynthesis [83]. Although the molecular mechanisms for the induction of anthocyanin synthesis by JA signaling have made great progress, the molecular regulatory networks of JA biosynthesis, especially how JA biosynthesis can be induced by wounding, remain largely unclear. Our comprehensive biochemical assays proved that wounding can greatly induce the transcription of *VvWRKY5* (Fig. 3a), and VvWRKY5 then binds directly to the *VvLOX* promoter to increase its activity, thereby promoting JA biosynthesis near the wounding site (Fig. 7). In addition, the interaction between VvWRKY5 and VvMYBA1 resulted in higher activation of the *VvLOX* promoter by VvWRKY5, indicating that the WRKY-MYB module is also critical in JA-induced anthocyanin biosynthesis. Interestingly, *cis*-element analysis revealed that both *VvWRKY5* and *VvMYBA1* promoters contained MeJA responsive elements (Fig. S6, see online supplementary material), and the expression levels of *VvWRKY5* and *VvMYBA1* were significantly induced after MeJA treatment (Fig. S7, see online supplementary material), indicating a possible feedback loop in wounding-induced anthocyanin. Wounding induced the biosynthesis of MeJA, and MeJA induced the expression of *VvWRKY5* and *VvMYBA1*. Furthermore, VvWRKY5 also promote the biosynthesis of MeJA.

**Figure 8:**
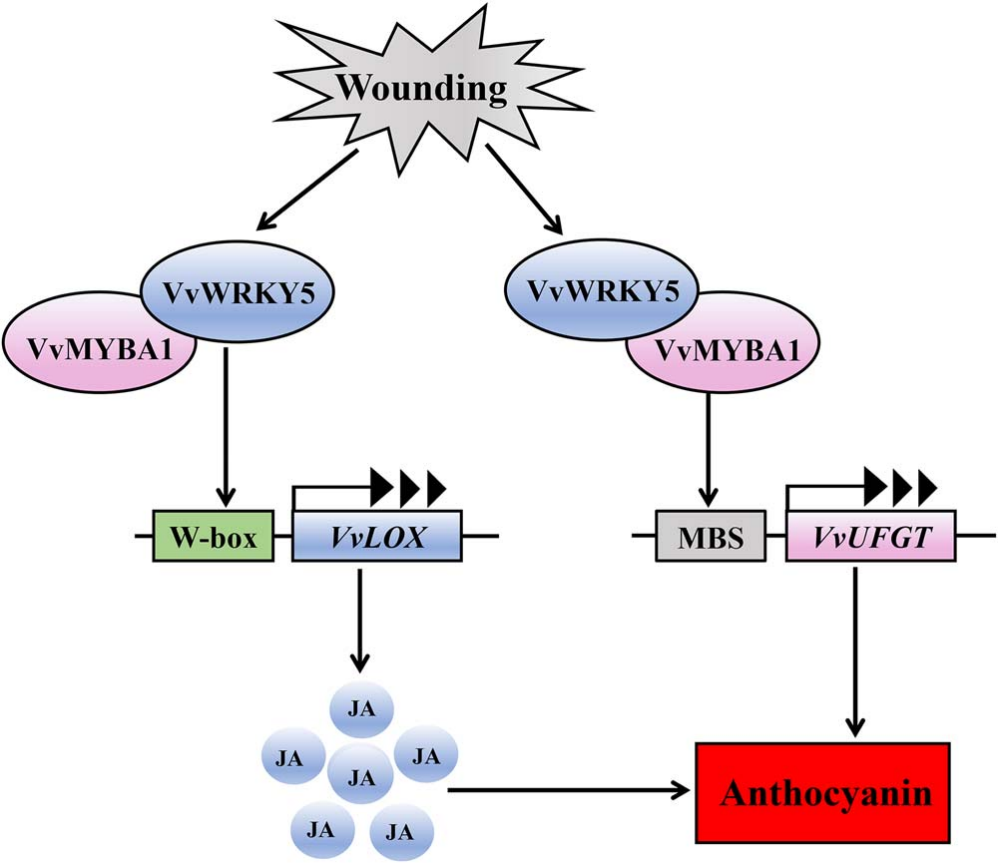
A possible model for the function of VvWRKY5 in wounding-induced anthocyanin deposition in grape. On the one hand, wounding induces the transcription of *VvWRKY5*, which protein combines with VvMYBA1 to amplify the transcriptional activation of VvMYBA1 on the *VvUFGT* promoter, thereby facilitating wounding-mediated anthocyanin synthesis. On the other hand, VvWRKY5 directly activates the transcription of *VvLOX* to boost the synthesis of JA, a phytohormone that plays a promotive role in grape anthocyanin production. JA, jasmonic acid.

Our data provide new insight into the possible mechanism of wounding-induced anthocyanin accumulation (Fig. 8). In short, VvWRKY5 is a positive modulator of wounding-induced anthocyanin production. In the presence of wounding, the expression level of *VvWRKY5* is elevated. On the one hand, VvWRKY5 interacts with VvMYBA1 and enhances VvMYBA1 activation on the *VvUFGT* promoter, thereby promoting anthocyanin synthesis. On the other hand, VvWRKY5 promotes the synthesis of JA, a phytohormone that functions as a positive modulator in the accumulation of grape anthocyanins, by directly linking to the *VvLOX* promoter to induce its expression, and this activation is also enhanced by VvMYBA1. Given the importance of anthocyanin in protecting plants from external stresses [65, 66] and the extensive stress responses of *VvWRKY5*, we speculate that VvWRKY5 may also be involved in the modulation of other stress responses, which requires further investigation in the future. Collectively, our findings contribute to a better understanding of the function of VvWRKY5 in wounding-induced anthocyanin production in grape.

## Conclusion

VvWRKY5 plays a positive regulatory role in grape wounding-induced anthocyanin synthesis. We found that VvWRKY5 can interact with VvMYBA1 to enhance VvMYBA1's activation of *VvUFGT* promoter, thereby promoting anthocyanin accumulation. In addition, VvWRKY5 can promote JA biosynthesis by directly binding to the *VvLOX* promoter to induce its expression, while VvMYBA1 can also enhance this activation. This study enriched the transcriptional regulation mechanism of wounding-induced anthocyanin accumulation.

## Materials and methods

### Plant materials, growth conditions, and treatments

The grape cultivar ‘Red Globe’ fruits were harvested at 110 DAFB from an orchard at Shenyang Agricultural University, Shenyang city, Liaoning Province, China, and utilized for the wounding and MeJA treatments. For the wounding treatment, the equatorial side of ‘Red Globe’ grape fruits was treated with a wooden stick, the fruit stalk was wrapped with absorbent cotton to reduce water loss, and the wounding-treated grape fruits were incubated in an incubator under constant light conditions (3000 lux light intensity, 22°C). Samples of the grape skins surrounding the wounds were taken for the anthocyanin determination and gene expression analysis. For the MeJA treatments, grape fruits were immersed with 100 μM MeJA solution for 1 min, dried naturally, and then incubated in an incubator with constant light. The anthocyanin determination and gene expression analysis were conducted after 7 days of MeJA treatment.

‘Gamay Freaux’ grape calli, which were utilized for genetic transformation and coloration experiments, were cultured on a solid B5 medium containing 0.2 mg·L^−1^ kinetin, 250 mg·L^−1^ casein hydrolysate, 100 mg·L^−1^ myoinositol, 2% sucrose, and 0.1 mg·L^−1^ NAA in an incubator (3000 lux light intensity, 25°C) and subcultured every 25 days [84].

The tobacco (*N. benthamiana*) utilized for transient expression experiments was grown in an incubator at 25°C with a period of 16 h light/8 h dark.

### Grape fruit transient transformation

The coding sequence (CDS) of *VvWRKY5* was ligated into the pRI101-AN vector to obtain the VvWRKY5-pRI overexpression vector, whilst the 393-bp *VvWRK5* CDS fragment and 390-bp *VvMYBA1* CDS fragment were cloned into the pTRV2 vectors to obtain the VvWRKY5-TRV and VvMYBA1-TRV suppression expression vectors. *Agrobacterium tumefaciens* EHA105 carrying the above recombinant constructs was used to inject ‘Red Globe’ grape fruits (110 DAFB) in different combinations. The infected grapes were incubated in an incubator under constant light conditions for 5 days at 22°C. Each treatment had three biological replicates, and 20 injected grape berries were used as one biological replicate. The relevant primers are listed in Table S1 (see online supplementary material).

### Stable genetic transformation of grape calli

VvWRKY5-OE grape calli were obtained as previously described [63]. The VvWRKY5-pRI plasmid was transferred into *A. tumefaciens* EHA105 via the freeze–thaw method. Two-week-old grape calli were incubated with plasmid-transformed *A. tumefaciens* at room temperature for 30 min. Then, the infected calli was transferred to antibiotic-free B5 medium for 2 days in the dark at room temperature. Finally, they were plated on B5 medium containing 40 mg/L paromomycin sulphate and 300 mg/L cefotaxime sodium in the light to select for transformants. VvWRKY5-OE/VvMYBA1-Anti grape calli were obtained by introducing the VvMYBA1-pFGC1008 recombinant vector into VvWRKY5-OE grape calli. The positive transgenic grape calli were detected by RT–qPCR and cultured in a phytotron under constant light conditions. The relevant primers are listed in Table S1 (see online supplementary material).

### Determination of total anthocyanin content

The methanol–HCl method was utilized for total anthocyanin extraction in grape [85]. Powdered grape peel or calli samples (0.2 g) were combined with 5 mL methanol containing 0.1% (v/v) HCl overnight in darkness at 4°C. The determination of total anthocyanin content in samples was accomplished using the pH differential method [86]. The absorbance of extracts from the samples was recorded at 520 and 700 nm utilizing a spectrophotometer (UV-2450, Kyoto, Japan). The results were displayed as milligram cyanidin-3-glucoside equivalents/100 g FW using a molecular weight of 449.2 and a molar absorptivity of 26 900. All experiments were repeated in triplicate.

### RT–qPCR assay

Total RNA was gathered from about 15 representative grape berries and reversed into single-stranded DNA as described previously [87]. RT–qPCR was conducted with the UltraSYBR Mixture on a 7500 Real-Time PCR equipment (Applied Biosystems, Foster City, CA, USA). Three biological and technical replicates were used for every sample. *VvActin* (XM_002278316.4) was served as an endogenous standard. The 2^-ΔΔCt^ approach was utilized to determine the level of gene expression [88]. The relevant primers utilized are provided in Table S2 (see online supplementary material).

### Y2H assays

The *VvMYBA1* coding region was ligated to the activation domain vector pGADT7, while the N-terminal of the *VvWRKY5* coding region was inserted into the DNA-binding domain vector pGBKT7. The recombinant vectors VvMYBA1-AD and VvWRKY5^N^-BD were co-transferred into the Y2H yeast cells. Transferred yeast strains were grown on SD selection medium (−T/−L/−H/−A) containing X-α-gal and cultured in a 30°C incubator for 3 days to investigate their interactions. The relevant primers utilized are provided in Table S1 (see online supplementary material).

### Pull-down assays

We fused the full-length *VvWRKY5* and *VvMYBA1* to the pET-32a and pGEX-4 T-1 vectors, respectively. The VvWRKY5-HIS- and VvMYBA1-GST-fused proteins were produced in BL21 cells (TransGen, Beijing, China). Proteins were purified employing a commercial kit (CWbio, Beijing, China) following the protocols provided by the manufacturer. Western blot (WB) tests were conducted to examine the pulled-down products with GST and HIS antibodies. Relevant primers utilized are provided in Table S1 (see online supplementary material).

### LCI assays

For LCI assays, the *VvMYBA1* CDS was ligated into the pCAMBIA1300-nLUC vector, while the *VvWRKY5* CDS was introduced into the pCAMBIA1300-cLUC vector. These recombinant constructions of VvWRKY5-cLUC and VvMYBA1-nLUC were translated into *A. tumefaciens* GV3101 cells. Then, *N. benthamiana* leaves were infiltrated with an Agrobacterium mixture (OD_600_ = 1) carrying the recombinant plasmids. Two days after infiltration, luciferase signaling was detected by a Tanon-5200 plant visualization device (Shanghai, China). The relevant primers utilized are provided in Table S1 (see online supplementary material).

### BiFC assays

The codon-free *VvWRKY5* CDS was connected to the 35S-pSPYNEY-YFP vector, and the complete *VvMYBA1* CDS was connected to the 35S-pSPYCEY-YFP vector. Thereafter, we introduced the recombinant plasmids VvWRKY5-YFP^N^ and VvMYBA1-YFP^C^ into *A. tumefaciens* strain GV3101. The onion epidermal cells were incubated with Agrobacterium suspension (OD_600_ = 1) carrying the recombinant plasmid. Two days after infection, fluorescence was detected by a Leica DMi8 A confocal microscope (Wetzlar, Germany). The relevant primers utilized are provided in Table S1 (see online supplementary material).

### Y1H assays

The VvWRKY5-AD prey plasmid was constructed as previously described [63]. To construct bait plasmids, the *VvMYBA1*, *VvUFGT*, *VvLOX*, and *VvOPR3* promoters were fused to pHIS2 vectors (*proVvMYBA1*-pHIS2, *proVvUFGT*-pHIS2, *proVvLOX*-pHIS2, and *proVvOPR3*-pHIS2). The Y187 yeast cells harboring specific combinations of prey and bait plasmids were grown in a 30°C incubator on SD/−Trp/−Leu/−His selection media adding 3-AT and X-α-gal for 3 days. The relevant primers utilized are provided in Table S1 (see online supplementary material).

### EMSA

EMSAs were conducted following previously described methods [89]. The cDNA sequences of *VvWRKY5* and *VvMYBA1* were integrated into the pET-32a vectors. The *Escherichia coli* BL21 cells carrying these recombinant vectors were induced at 16°C for 24 h to obtain His-tagged fusion proteins (VvWRKY5-HIS and VvMYBA1-HIS). A Ni-agarose His-Tag Purification Kit (CWbio, Beijing, China) was utilized for protein purification. EMSAs were carried out with the LightShift Chemiluminescent EMSA system to detect the interaction of the VvMYBA1-HIS protein with the *VvUFGT* promoter or the VvWRKY5-HIS protein with the *VvLOX* promoter. In addition, the VvWRKY5-HIS protein was added to investigate if VvWRKY5 influenced the binding of VvMYBA1-HIS to the *VvUFGT* promoter. The relevant probes utilized are provided in Table S3 (see online supplementary material).

### ChIP-PCR analysis

For ChIP-PCR experiments, the *VvWRKY5* CDS was connected to the GFP-tagged pRI101 vector (VvWRKY5-GFP) and transformed into *A. tumefaciens* EHA105 cells. Twenty-day-old ‘Gamay’ grape calli were prepared for stable transformation. An EZ-ChIP Chromatin Immunoprecipitation Kit (Millipore, Waltham, MA, USA) was utilized for ChIP experiments, and PCR was utilized to detect the enriched DNA fragments. The relevant primers utilized are provided in Table S1 (see online supplementary material).

### Dual-luciferase reporter assays

The *VvUFGT* and *VvLOX* promoter fragments (~2000 bp upstream of ATG) were fused to the pGreenII0800-LUC vectors to produce the reporters (*proVvUFGT*-LUC and *proVvLOX*-LUC). The *VvMYBA1* and *VvWRKY5* CDSs were fused to the pRI101-AN vectors to produce the effectors (35S::*VvMYBA1* and 35S::*VvWRKY5*). These recombinant constructs were introduced into Agrobacterium GV3101. Next, a mixed Agrobacterium suspension (OD_600_ = 1) harboring the recombinant plasmids was transiently injected into *N. benthamiana* leaves. These tobacco plants were then grown in dark conditions. Two days after infection, luciferase signaling was detected by a Tanon-5200 imaging device (Shanghai, China), while promoter activity based on the LUC/REN ratio was assessed employing a luciferase detection kit (Beyotime, Shanghai, China). Relevant primers utilized are provided in Table S1 (see online supplementary material).

### Statistical analysis

Every test was performed with three separate biological replicates. Microsoft Excel 2021 and GraphPad Prism 6 were utilized for data analysis. Different letters or asterisks represent statistical significance detected by Tukey’s test utilizing DPS 9.01 software.

## Acknowledgements

We are grateful to Dr Haifeng Jia at Nanjing Agricultural University for providing the ‘Gamay’ grape calli. The work was funded by the China Agriculture Research System (CARS-29-yc-6), the National Natural Science Foundation of China (31972368), the Major Agricultural Science Projects of Liaoning Province (2023JH1/10200004), and the Science and Technology Program of Shenyang (23-410-2-03).

## Author contributions

Z.Z. and Y.G. conceived the experiments. Z.Z. and C.C. performed the experiments. H.L. and C.J. assisted with the research. Z.Z., C.C., and Y.Z. analysed the data. Z.Z. and Y.G. wrote and modified the article.

## Data availability

Data supporting the results are available in the article and its supplementary data.

## Conflict of interest statement

The authors declare no competing interests.

## Supplementary data

Supplementary data is available at *Horticulture Research* online.

## Supplementary Material

Web_Material_uhae083
