# Supplementary material for: VvWRKY5 positively regulates wounding-induced anthocyanin accumulation in grape by interplaying with VvMYBA1 and promoting jasmonic acid biosynthesis
Source: Hortic Res. 2024 Mar 25;11(5):uhae083. doi: 10.1093/hr/uhae083 (PMC11101322; doi:10.1093/hr/uhae083)
Supplement: Web_Material_uhae083 [file web_material_uhae083.zip › Supplementary Data.docx]

**
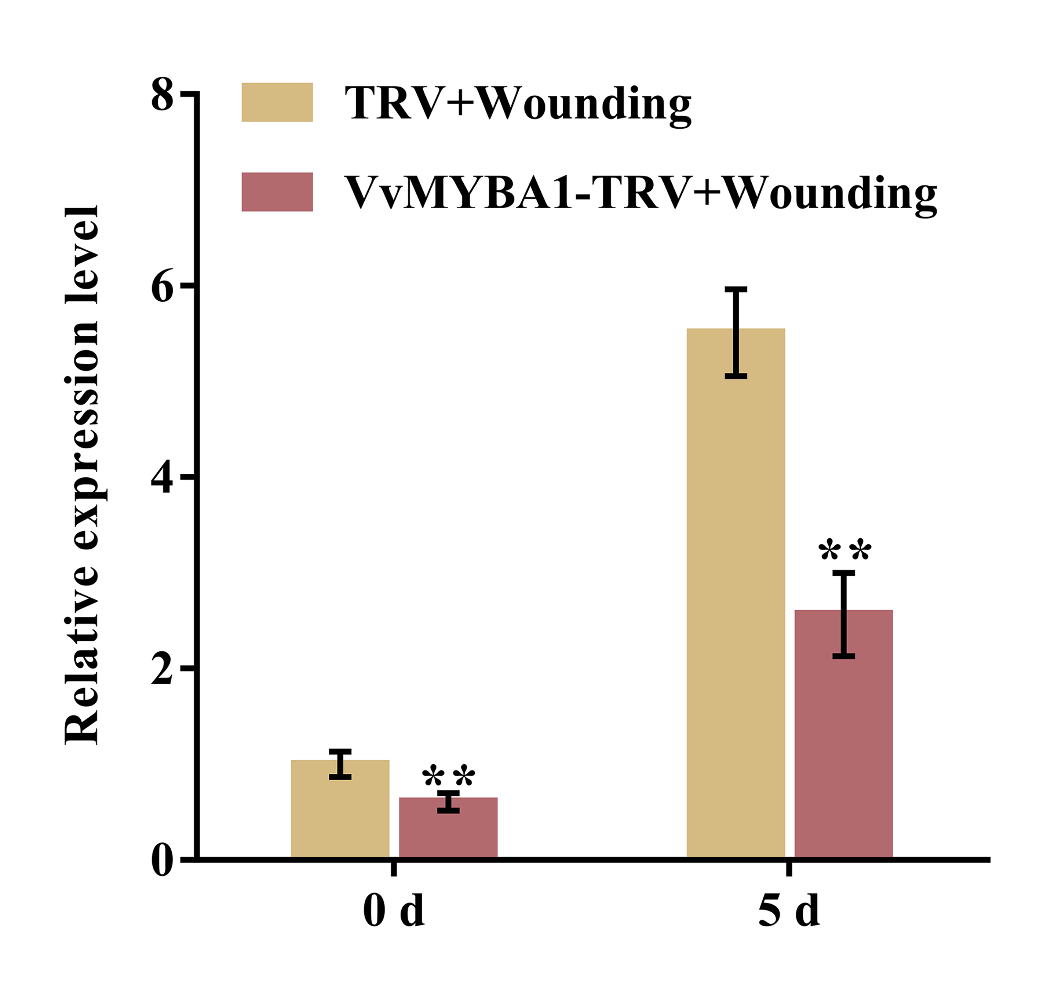
**

**Supplementary Figure S1.** Relative expression level of *VvMYBA1* in TRV and VvMYBA1-TRV ‘Red Globe’ grape fruits after wounding treatment. Data represent the means ± SDs of three separate biological replicates. The statistical significance was determined by Tukey’s test (***P* < 0.01).


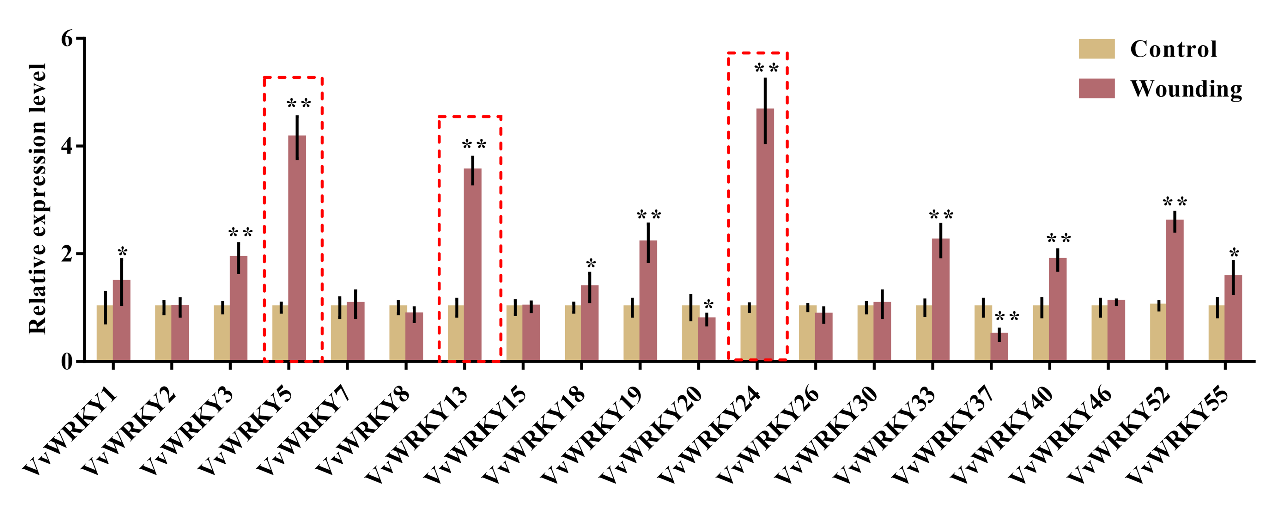


**Supplementary Figure S2.** RT‒qPCR expression profiles of 20 selected WRKY genes in wounded grape fruits. *VvActin* was used as an internal control gene. Data represent the means ± SDs of three separate biological replicates. The statistical significance was determined by Tukey’s test (**P* < 0.05 and ***P* < 0.01).


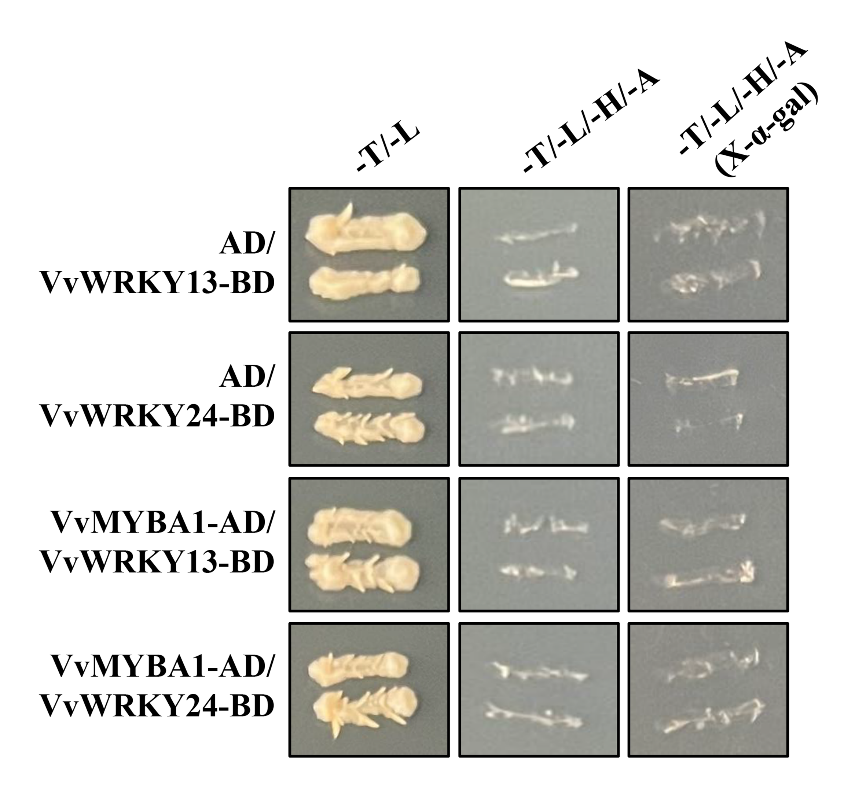


**Supplementary Figure S3.** VvWRKY13 and VvWRKY24 did not interact with VvMYBA1 in Y2H assays.


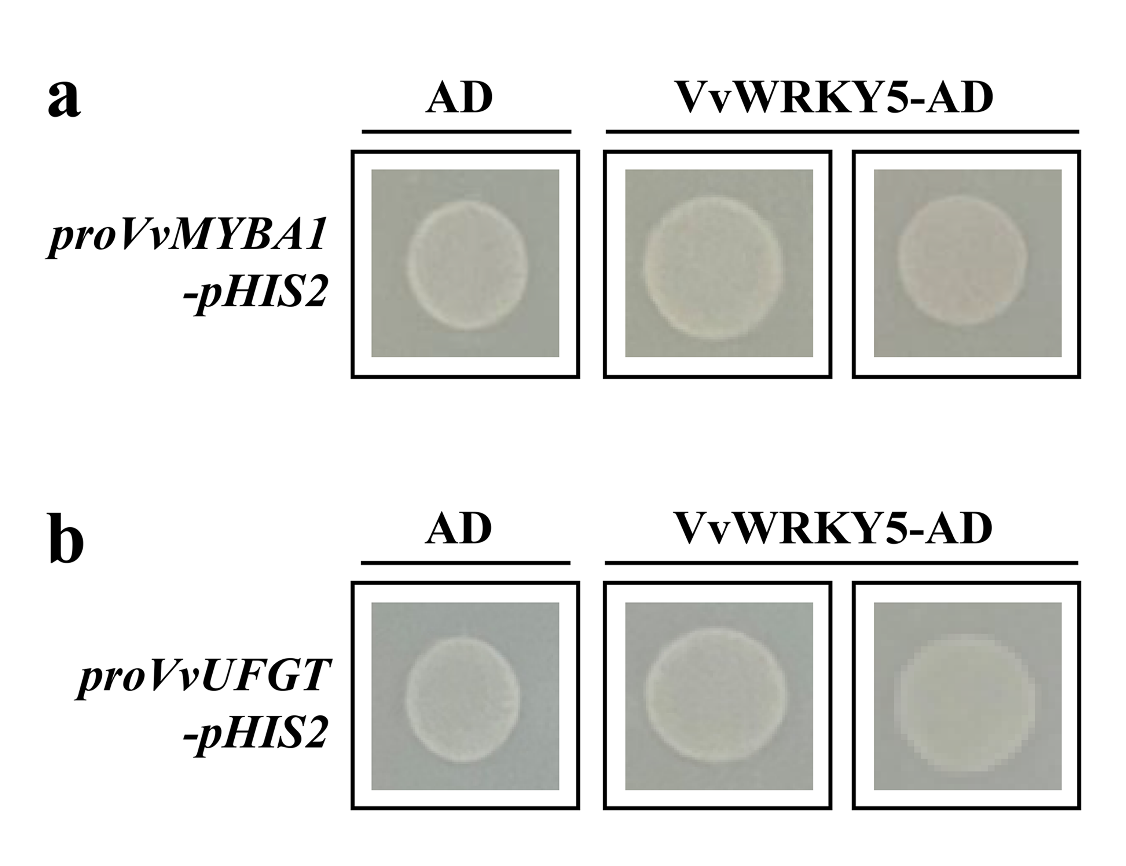


**Supplementary Figure S4.** YIH assays showed that VvWRKY5 did not interact with the *VvMYBA1* (**a**) and *VvUFGT* (**b**) promoters.


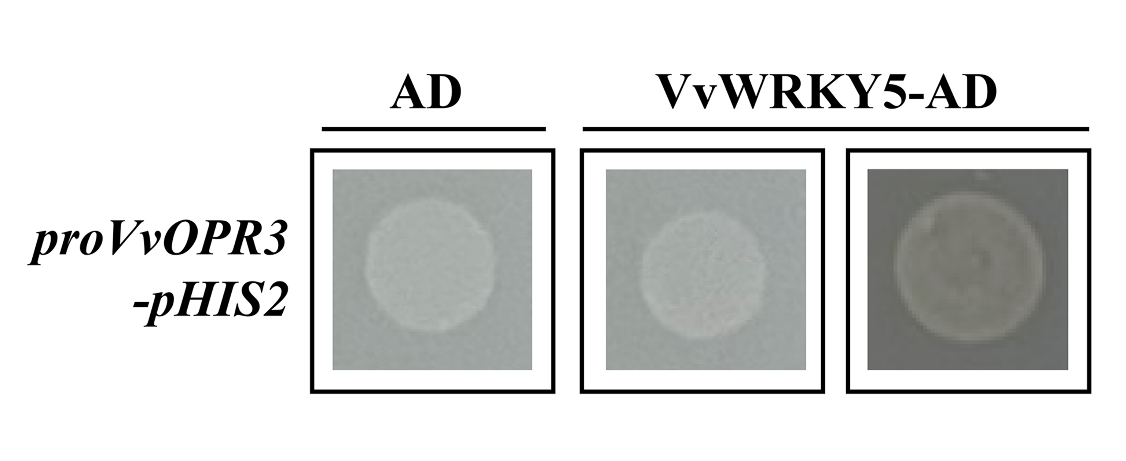


**Supplementary Figure S5.** YIH assays showed that VvWRKY5 did not interact with the *VvOPR3* promoter.


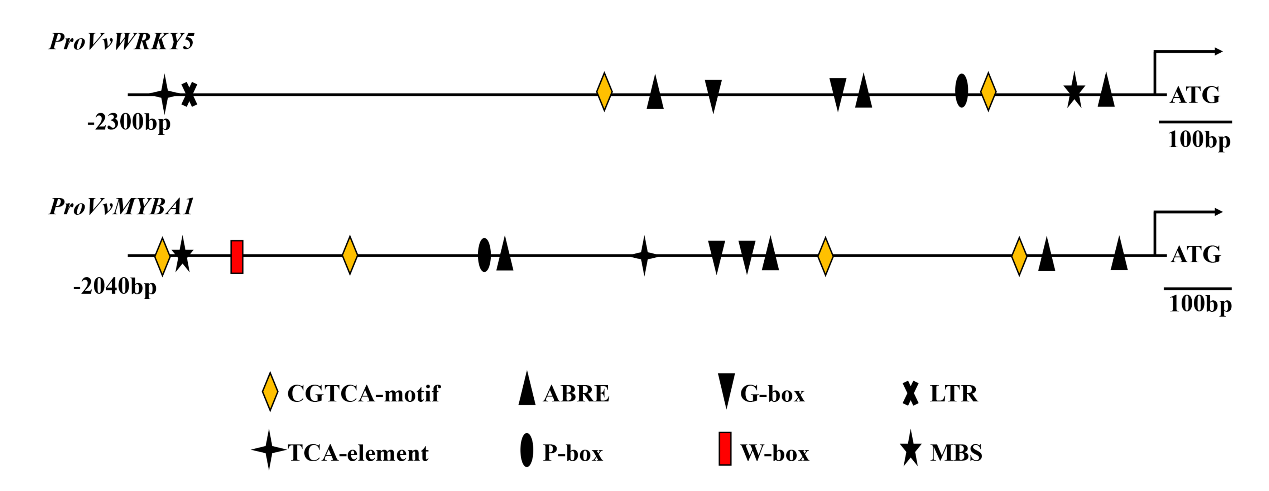


**Supplementary Figure S6.** Cis-element analysis of the *VvWRKY5* and *VvMYBA1* promoters.


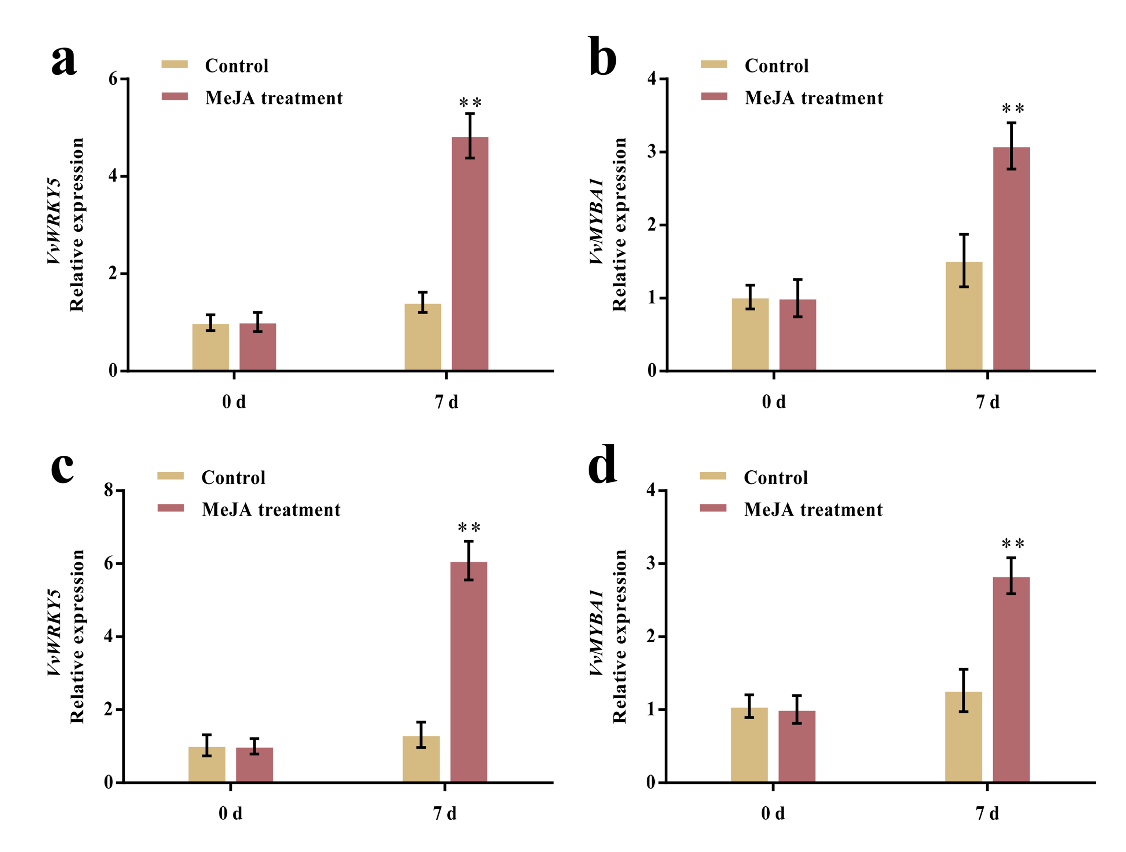


**Supplementary Figure S7.** Expression profiles of *VvWRKY5* and *VvMYBA1* under MeJA treatment. **a**, **b** RT‒qPCR analysis of the expression patterns of *VvWRKY5* and *VvMYBA1* in grape fruits under MeJA treatment. **c**, **d** RT‒qPCR analysis of the expression patterns of *VvWRKY5* and *VvMYBA1* in grape calli under MeJA treatment. Data are means ± SDs of 3 separate experiments. Statistical significance at *P* < 0.01 (**) was detected using Tukey’s test.

**Supplementary Table S1. Primers used for vector construction**

| **Gene Name** | **Forward (5′-3′)** | **Reverse (5′-3′)** |
| --- | --- | --- |
| Transgene | | |
| VvMYBA1-TRV2 | GAATTCAACAGATGGTCCTTGATTGC | CCCGGGGAGTTTCTTCTGTCCTGAGGC |
| VvMYBA1-pFGC1008 | AGGCGCGCCAACAGATGGTCCTTGATTGC | ATTTAAATAGTTTCTTCTGTCCTGAGGC |
| VvMYBA1-pFGC1008 | GGACTAGTAACAGATGGTCCTTGATTGC | CGGGATCCAGTTTCTTCTGTCCTGAGGC |
| VvWRK5-TRV2 | GAATTCGGAGTTATTACAGGTGCAGTAG | CCCGGGCATCAGAGATGTGAACCTCGT |
| VvWRKY5-pRI101 | GTCGACATGGGGGACTGGGATTTGCAG | GGATCCGCAACCGCCGGCGGCAGTTG |
| Y1H assay | | |
| VvWRKY5-pGADT7 | CATATGATGGGGGACTGGGATTTGCA | GGATCCTCAGCAACCGCCGGCGGC |
| *proVvUFGT*-pHIS2 | GAATTCTAAACAAGGTGAGACGAGATGG | GAGCTCGGTTGGAATGGGGGATGT |
| *proVvLOX*-pHIS2 | GAATTCCCCTCAAGCTATGCCATGA | GAGCTCGCTCAGGTGGTGCATGTATT |
| *proVvOPR3*-pHIS2 | GAATTCGGTGTACAAAATTTCTAACGTGG | GAGCTCTGTCGTCTACGAGTCCGGT |
| ChIP-PCR assay | | |
| VvWRKY5-pRI101-GFP | GTCGACATGGGGGACTGGGATTTGC | GGATCCGCAACCGCCGGCGGCAGTTG |
| *proVvLOX*-W-box | CCAACCAGCCAATGGAAA | GCTCAGGTGGTGCATGTAT |
| Dual-luciferase reporter assay | | |
| VvWRKY5-pRI101 | GTCGACATGGGGGACTGGGATTTGC | GGATCCGCAACCGCCGGCGGCAGTTG |
| VvMYBA1-pRI101 | GTCGACATGGAGAGCTTAGGAGTTAGAAAG | GAATTCGATCAAGTGATTTACTTGTGTGTTG |
| *proVvUFGT*-pGreenII0800-LUC | AAGCTTTAAACAAGGTGAGACGAGATGG | GGATCCGGTTGGAATGGGGGATGT |
| *proVvLOX*-pGreenII0800-LUC | AAGCTTCCCTCAAGCTATGCCATGA | GGATCCGCTCAGGTGGTGCATGTATT |
| Y2H assay | | |
| VvMYBA1-pGADT7 | GAATTCATGGAGAGCTTAGGAGTTAGAAAG | GAGCTCTCAGATCAAGTGATTTACTTGTGTG |
| VvWRKY5^N^-pGBKT7 | CCCGGGTCTCATCCAACTCGCCGGAA | GTCGACTCAGCAACCGCCGGCGGC |
| VvWRKY13-pGBKT7 | CCCGGGATGGCCAGGAGCTTGGATGACC | GTCGACTCAAGAAAAAGGAGATTGGGA |
| VvWRKY24-pGBKT7 | CCCGGGATGGACTTCCCACCACCCCT | GTCGACTCAATCCGTGGCGGTGTAAC |
| Pull-down assay | | |
| VvWRKY5-PET32a | GGATCCATGGGGGACTGGGATTTGCA | GTCGACTCAGCAACCGCCGGCGGC |
| VvMYBA1-PGEX4T-1 | GAATTCATGGAGAGCTTAGGAGTTAGAAAG | GTCGACTCAGATCAAGTGATTTACTTGTGTG |
| BiFC assay | | |
| VvMYBA1-YFP^C^ | GTCGACATGGAGAGCTTAGGAGTTAGAAAG | CCCGGGTCAGATCAAGTGATTTACTTGTGTG |
| VvWRKY5-YFP^N^ | GGATCCATGGGGGACTGGGATTTGCA | GTCGACGCAACCGCCGGCGGCAGTTG |
| LCI assay | | |
| VvWRKY5-cLUC | GGATCCATGGGGGACTGGGATTTGCA | CTCGAGTCAGCAACCGCCGGCGGC |
| VvMYBA1-nLUC | GAGCTCATGGAGAGCTTAGGAGTTAGAAAG | GTCGACGATCAAGTGATTTACTTGTGTGTTG |

**Supplementary Table S2. Primers used for RT‒qPCR**

| **Gene Name** | **Forward (5′-3′)** | **Reverse (5′-3′)** |
| --- | --- | --- |
| VvActin | GATTCTGGTGATGGTGTGAGT | GACAATTTCCCGTTCAGCAGT |
| VvWRKY1 | GACCCCTCCATTAACATCACA | CCCAAGCAGTCCTTCATCAGT |
| VvWRKY2 | TATGGGGCACTGTCGTCCT | GTTGCGGTGGCTATGGTC |
| VvWRKY3 | AGTTCCCGCCTCAGACATC | TTTTGCCCATACTTCCTCC |
| VvWRKY5 | TGTGGGCTTGGCGTAAATA | GGCGAGTTGGATGAGAATGG |
| VvWRKY7 | AGGAAATACGGGCAAAAG | GCAGAACAGAGTCCCACAAA |
| VvWRKY8 | ATTCCTGGCATCCGAGTTT | CTTGGATTTGGGCTGTTCTT |
| VvWRKY9 | CGGGACGGGTCAGGAGAAG | CGCAGCAGCCAAAGCAAG |
| VvWRKY13 | GCCAAGTGAAGAAGAGGATAGA | AAGGAGATTGGGAGGAAGC |
| VvWRKY15 | TGCTGATCCCCACCAACC | CTGCTGCCCCACGAAATA |
| VvWRKY18 | TGGCAGTTACCACGCATCA | CTCGGGTTTTCGCTTCCTT |
| VvWRKY19 | GGGGAGGCTGTGGTTAGGTT | GTTTGGCATTTGGCTTGTCT |
| VvWRKY20 | GGTCTTGGAGGAAATATGGG | TAGGCTGTTGGGAGTGGT |
| VvWRKY24 | TTTGAACCCTCCGACTACC | CTTCGCCCCATCTTTACAT |
| VvWRKY26 | GACCTCTTGGACTCCCCTC | AAGCCTGCTGGTGCTCGTT |
| VvWRKY33 | TTTGGCTTGTCACCTTCATCC | CGGTCCTTCTTACTCTTGCTT |
| VvWRKY37 | CAGTCAAGAATAGCCCTTACCC | CAGCATTGCCTCTTATCGTAG |
| VvWRKY40 | ACCAAATCCTTCCATCATCAC | TGCAGCCAAGTCAGTCCTAG |
| VvWRKY46 | TACCATCAGACCCTAAGAGTTCC | TGTCCATATTTCCGCCAGTT |
| VvWRKY52 | CCTCTTGATGATGGGTTTAGTT | GTCTTCCACGGTAGGTGATTT |
| VvWRKY55 | GGAGACACCGAAAGCGT | AATGACACCGCCCAAAAG |
| VvMYBA1 | AAGCCATCATCCACTTCA | GCGATAAGCATCTCTCCA |
| VvUFGT | GGGATGGTAATGGCTGTGG | ACATGGGTGGAGAGTGAGTT |
| VvCHS | CAAGACATGGTGGTGGTTG | TTGACGGAGGGTTTGAGGC |
| VvCHI | CAGGCAACTCCATTCTTTTC | TTCTCTATCACTGCATTCCC |
| VvANS | GTTCATAGGGCGGTTTGTG | GAGGGACTTGATGGTGCTG |
| VvDFR | GAAACCTGTAGATGGCAGGA | GGCCAAATCAAACTACCAGA |
| VvF3H | CCAATCATAGCAGACTGTCC | TCAGAGGATACACGGTTGCC |
| VvLOX | GAGCCTACCACATCCTAAT | GTCATTCACAGCAGCATAA |
| VvAOS | TTCTTGCTGGCTTCAATG | GCTTTAACAACGGTCCTTAT |
| VvOPR3 | GGCAGTGAAGAAGAGGAA | GCGACCATAGGATACCAA |
| VvOPCL1 | GCCTTGTTACTTACTCACCCAGA | GATCCTTCCTCAGAATCTTGCCT |
| VvAOC | GCGTGCTGATACAGAATG | TATCCTCGTAGGTCAAGTAAG |

**Supplementary Table S3. Primers used to synthesize probes for EMSA**

| **Gene Name** | **Forward (5′-3′)** | **Reverse (5′-3′)** |
| --- | --- | --- |
| VvUFGT-Biotin Probe | GGATGACAACCCCCATGCAGTTGCCACTCTCACAACCCCC | GGGGGTTGTGAGAGTGGCAACTGCATGGGGGTTGTCATCC |
| VvUFGT-Mutant Probe | GGATGACAACCCCCATGCGGTCGCCACTCTCACAACCCCC | GGGGGTTGTGAGAGTGGCGACCGCATGGGGGTTGTCATCC |
| VvLOX-Biotin Probe | ACCAGCCAATGGAAAATAGTCAAAATACCTAGTCTATTCC | GGAATAGACTAGGTATTTTGACTATTTTCCATTGGCTGGT |
| VvLOX-Competitive Probe | ACCAGCCAATGGAAAATAGTCAAAATACCTAGTCTATTCC | GGAATAGACTAGGTATTTTGACTATTTTCCATTGGCTGGT |
| VvLOX-Mutant Probe | ACCAGCCAATGGAAAATAGCGAAAATACCTAGTCTATTCC | GGAATAGACTAGGTATTTTCGCTATTTTCCATTGGCTGGT |
